# Quantitative aspects of nitric oxide production from nitrate and nitrite

**DOI:** 10.17179/excli2022-4727

**Published:** 2022-02-21

**Authors:** Asghar Ghasemi

**Affiliations:** 1 Endocrine Physiology Research Center, Research Institute for Endocrine Sciences, Shahid Beheshti University of Medical Sciences, Tehran, Iran

**Keywords:** nitric oxide, nitrate, nitrite, rate of nitric oxide production

## Abstract

Nitric oxide (NO) is involved in many physiological and pathological processes in the human body. At least two major pathways produce NO: (1) the *L*-arginine-NO-oxidative pathway in which NO synthase (NOS) enzymes convert *L*-arginine to NO; (2) the nitrate-nitrite-NO reductive pathway in which NO is produced from the serial reduction of nitrate and nitrite. The deficiency of NO is involved in the pathophysiology of cardiometabolic disorders. Intervention with foods containing nitrate and nitrite can potentially prevent or treat some chronic diseases, including cardiovascular diseases and diabetes. A better understanding of the NO cycle would help develop effective strategies for preventing or treating the disorders in which NO homeostasis is disturbed. This review summarizes quantitative aspects of NO production, emphasizing the nitrate-nitrite-NO pathway. Available data indicates that total NO production by NOS-dependent *L*-arginine-NO pathway is about 1000 μmol.day^-1^. Of about 1700 μmol.day^-1 ^ingested nitrate, ~25 % is extracted by the salivary glands and of which ~20 % is converted nitrite. It means that about 5 % of ingested nitrate is converted to nitrite in the oral cavity; assuming that all produced nitrite is reduced to NO in the stomach, it can be calculated that contribution of the nitrate-nitrite-NO pathway to the whole-body NO production is about 85 μmol.day^-1 ^(1700 ×0.05=85) or approximately 100 μmol.day^-1^. The lower contribution of the nitrate-nitrite-NO pathway does not mean that this pathway has lower importance in the whole-body NO homeostasis. Even in the adequate *L*-arginine supply, NOS-dependent NO production is insufficient to meet all NO functions, and the nitrate-nitrite-NO pathway must provide the rest. In conclusion, the contribution of the nitrate-nitrite-NO pathway in the whole human body NO production is <10 %, and the nitrate-nitrite-NO pathway is complementary to the NOS-dependent NO production.

## Introduction

In 1980, it was reported that acetylcholine stimulates endothelial cells to release a substance, which causes relaxation of vascular smooth muscle cells (Furchgott and Zawadzki, 1980[25]). Two years later, the substance was called endothelium-derived relaxing factor (EDRF) (Cherry et al., 1982[19]). After five years, it was identified that EDRF is nitric oxide (NO) (Furchgott, 1987[24]; Ignarro et al., 1987[36]; Palmer et al., 1987[79]). In 1988, *L*-arginine was identified as the endogenous substrate for NO synthesis (Palmer et al., 1988[78]; Schmidt et al., 1988[88]). After that, NO synthase (NOS) enzymes were isolated, including neural NOS (nNOS or NOS I) (Bredt and Snyder, 1990[11]), inducible NOS (iNOS or NOS II) (Hevel et al., 1991[32]), and endothelial NOS (eNOS or NOS III) (Pollock et al., 1991[81]). These findings led to the discovery of the *L*-arginine oxidative pathway as the classic pathway of NO production in the human body. 

In 1994, NOS-independent NO generation was discovered in the stomach (Benjamin et al., 1994[7]; Lundberg et al., 1994[62]) and led to the introduction of the nitrate (NO_3_^-^)-nitrite (NO_2_^-^)-NO pathway as an alternative/backup system for the *L*-arginine-NO pathway (Lundberg and Weitzberg, 2010[60]). Nitrate and nitrite were previously thought to be environmental pollutants (Gladwin et al., 2005[30]) with potential carcinogenic effects (Lundberg et al., 2008[61]) or considered as inert by-products of the NO metabolism (Lundberg et al., 2008[61]). Despite existing controversies (Song et al., 2015[96]; Zhang et al., 2019[120]), no causal relationship between dietary intake of nitrate and nitrite and the risk of stomach cancer has been found (Bryan et al., 2012[13]). On the other hand, protective effects of nitrate and nitrite against cardiometabolic diseases have been highlighted as reviewed elsewhere (Ghasemi and Jeddi, 2017[27]; Kapil et al., 2020[46]; Lundberg et al., 2018[58]; Omar et al., 2016[76]; van Faassen et al., 2009[110]). Intervention by nitrate and nitrite is now one strategy for enhancing NO signaling because of their NO-like bioactivity (Lundberg et al., 2015[59]). These findings shifted the attention from toxic to favorable NO effects produced in the nitrate-nitrite-NO pathway.

The nutritional aspects of the nitrate-nitrite-NO pathway are of great importance as nitrate and nitrite, as oxidative products of NO, can also act as sources of NO generation (Li et al., 2003[53]). Dietary nitrate intake (Archer, 2002[1]), particularly vegetable consumption (Lidder and Webb, 2013[55]), is the major source of nitrate. Vegetable consumption is associated with decreased risk of metabolic syndrome (Tian et al., 2018[104]), type 2 diabetes (Carter et al., 2010[17]), total mortality (Miller et al., 2017[64]), stroke (Joshipura et al., 1999[45]), and other chronic diseases as reviewed elsewhere (Boeing et al., 2012[9]; Hung et al., 2004[34]). In addition, the beneficial cardiometabolic effects of some dietary interventions, such as the dietary approach to stop hypertension (DASH) diet, is attributed to its high contents of nitrate and nitrite (Ghasemi and Jeddi, 2017[27]). Therefore, intervention with foods containing nitrate and nitrite can prevent or treat some chronic diseases, including cardiovascular diseases and diabetes (Ghasemi and Jeddi, 2017[27]; Kapil et al., 2020[46]; Lundberg et al., 2018[58]; Omar and Webb, 2014[75]; Omar et al., 2016[76]; van Faassen et al., 2009[110]). 

A better understanding of the NO cycle would help develop effective strategies for preventing or treating the diseases in which NO homeostasis is disturbed. Despite reviews on NO synthesis by *L*-arginine-NO (Moncada and Higgs, 1993[68]; Solomonson et al., 2003[95]) and nitrate-nitrite-NO (Kapil et al., 2020[46], Lundberg et al., 2008[61]; McNally et al., 2016[63]) pathways, quantitative aspects of NO production have not been reviewed. This review highlights the relative contributions of these pathways in the whole-body NO production and NO production by the nitrate-nitrite-NO pathway. 

### Pathways of NO production 

**Figure 1:**
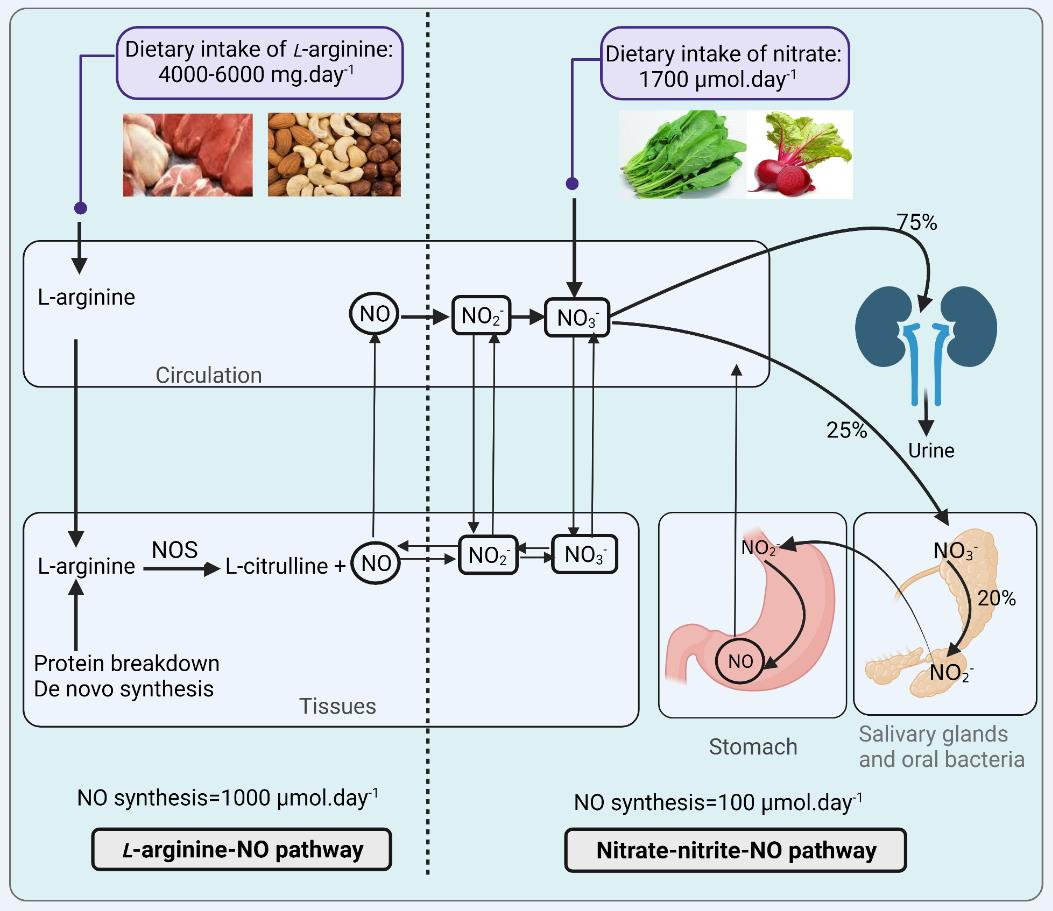
Pathways of nitric oxide (NO) production. NOS, NO synthase; NO_3_^-^, nitrate; NO_2_^-^, nitrite. Created with BioRender.com

At least two major pathways produce NO: (1) the *L*-arginine-NO-oxidative pathway; (2) the nitrate-nitrite-NO reductive pathway. Data on *L*-arginine, nitrate, and nitrite kinetics are presented in Table 1(Tab. 1) (References in Table 1: Archer, 2002[1]; Aschebrook-Kilfoy et al., 2013[2]; Babateen et al., 2018[3]; Bahadoran et al., 2019[4], 2021[6]; Bode-Böger et al., 1998[8]; Böger et al., 1997[10]; Calvert and Lefer, 2010[16]; Dejam et al., 2007[21]; EFSA, 2008[22]; Eriksson et al., 2018[23]; Hunault et al., 2009[33]; Inoue-Choi et al., 2016[40]; Kapil et al., 2020[46]; Lidder and Webb, 2013[55]; Lortie et al., 2000[57]; Mirmiran et al., 2017[67]; Morris, 2007[71]; Omar and Webb, 2014[75]; Packer and Leach, 1991[77]; Reinders et al., 2007[84]; Rougé et al., 2007[86]; Scientific Committee for Food, 1997[89]; Sheffner et al., 1948[91]; Simell and Perheentupa, 1974[93]; van Haeften and Konings, 1989[111]; van Velzen et al., 2008[112]; Wagner et al., 1983[114]; WHO, 2008[116]), and Figure 1(Fig. 1) summarizes NO production by these two pathways. In the *L*-arginine-NO pathway, NOS enzymes convert *L*-arginine to NO and *L*-citrulline in equimolar amounts (Lundberg et al., 2015[59]). *L*-arginine has both endogenous (de novo synthesis and protein turnover) and exogenous (i.e., diet) sources (Bahadoran et al., 2021[6]). Three different NOS isoforms have been identified, including constitutive (eNOS and nNOS) and inducible (iNOS) isoforms. 

In the nitrate-nitrite-NO pathway, NO is produced from the serial reduction of nitrate and nitrite (Lundberg et al., 2015[59]). Nitrate has endogenous (oxidation of NOS-derived NO and direct generation by NOS during the futile cycle) and exogenous (diet, water, and environment) sources (Kapil et al., 2020[46]; Lundberg et al., 2015[59]; Stuehr et al., 2004[101]). Food contributes about 80 % in the exogenous nitrate intake (Archer, 2002[1]), and about 85 % of dietary nitrate is derived from vegetables (Lidder and Webb, 2013[55]). Nitrate levels in vegetables vary widely (1-10,000 mg.kg^-1^) (Joint FAO/WHO Expert Committee on Food Additives, 1995[44]), and therefore, nitrate intake varies considerably between subjects. Two major sources of nitrite are oxidation of NOS-dependent NO generation and biotransformation of ingested nitrate (Jansson et al., 2008[42]). In physiological conditions, about 70 % of circulating and stored nitrite is derived from the oxidation of NOS-derived NO and the remainder 30 % from dietary intake of nitrate or nitrite (Omar and Webb, 2014[75]). Nitrite level in most foods is low (<10 mg.kg^-1^) (Joint FAO/WHO Expert Committee on Food Additives, 1995[44]), and therefore, ingested nitrite from dietary sources is very low (Packer and Leach, 1991[77]). In this pathway, nitrate reduction to nitrite and nitrite reduction to NO are achieved by bacteria (mainly in the oral cavity and to a lesser extent in the intestine) and mammalian cells (Jansson et al., 2008[42]).

### Whole-body NO production

Castillo et al. studied the whole-body NOS-dependent NO synthesis in healthy men (age: 23 ± 3 years) who received adequate arginine and protein intake and were on a low nitrate diet for 6 days (average daily nitrate intake of 210 μmol) (Castillo et al., 1996[18]). They found that rates of whole-body NOS-dependent NO synthesis in the human body are 1.00±0.2 and 0.91±0.3 μmol.kg^-1^.h^-1 ^in the fasted and fed conditions (average: 0.96±0.1 μmol.kg^-1^.h^-1^) using the conversion of plasma *L*-arginine to *L*-citrulline and 0.67±0.05 and 1.23±0.2 μmol.kg^-1^.h^-1 ^in the fasted and fed conditions (average: 0.95±0.1 μmol.kg^-1^.h^-1^) using the conversion of plasma *L*-arginine to urinary nitrate. On the other hand, in healthy subjects (13 male and 1 female, age 22-28 years), fasted NO synthesis (based on labeled *L*-arginine-to-*L*-citrulline conversion) has been reported to be 0.22 ± 0.07 μmol.kg^-1^.h^-1^ (Lagerwerf et al., 1998[50]). 

Results of a systematic review indicate that, in healthy subjects, the average rate of NOS-dependent NO synthesis depends on the method of measurement and is 0.74 ± 0.69 μmol.kg^-1^.h^-1^ using the arginine-to-citrulline protocol, 0.59 ± 0.29 μmol.kg^-1^.h^-1^ using the arginine-nitrate protocol, and 0.38 ± 0.06 μmol.kg^-1^.h^-1^ using the oxygen-nitrate protocol; pooling all studies, the average physiologic rate of NO synthesis in healthy subjects is 0.63±0.45 μmol.kg^-1^.h^-1^ (range: 0.22-1.23 μmol.kg^-1^.h^-1^). Assuming a 70-kg person, the rate of NOS-dependent NO production in healthy subjects is about 1000 μmol.day^-1^ (range: ~400-2000 μmol.day^-1^) (Siervo et al., 2011[92]). In support, following exclusion of dietary nitrate, about 1000 μmol.day^-1^ nitrate is excreted in the human urine, derived from the oxidation of NOS-derived NO (Walker, 1996[115]). Therefore, the contribution of endogenous nitrate to overall nitrate exposure in healthy humans is 900-1280 μmol.day^-1^ (Packer and Leach, 1991[77]) or about 1000 μmol.day^-1^ (Scientific Committee for Food, 1997[89], WHO, 2008[116]). 

Urinary nitrate excretion in eNOS^-/-^, nNOS^-/-^, and iNOS^-/-^ mice is decreased by 58 %, 39 %, and 47 %, respectively (Morishita et al., 2005[70]). Intravenous infusion of *L*-NMMA (N^G^-monomethyl-*L*-arginine) decreased NO synthesis in healthy subjects by 66 % (0.3 ± 0.14 vs 0.10 ± 0.06 μmol.kg^-1^.h^-1^) (Lagerwerf et al., 1998[50]). In triply n/i/eNOS^-/-^ mice, urinary NOx excretion decreased by 96-97 % compared to control mice, indicating the major role of the NOS-dependent pathway of NO production (Morishita et al., 2005[70]; Tsutsui et al., 2006[107]). 

Assuming that about 5 % of ingested nitrate is converted to nitrite in the oral cavity and that all of the nitrite is reduced to NO in the stomach, it can be calculated that the contribution of the nitrate-nitrite-NO pathway to the whole-body NO production is about 85 μmol.day^-1 ^(1700 × 0.05=85) or approximately 100 μmol.day^-1^. 

Overall, total NO production by NOS-dependent *L*-arginine-NO and nitrate-nitrite-NO pathways are about 1000 μmol.day^-1^ and 100 μmol.day^-1^, respectively, indicating <10 % contribution of the nitrate-nitrite-NO pathway in the whole human body NO production.

### Exposure to nitrate and nitrite 

According to a systematic review, median nitrate intake in 51 studies that included healthy subjects was 108 mg.day^-1^ (~1700 μmol.day^-1^) (Babateen et al., 2018[3]). However, total dietary exposure to nitrate ranges from 58 to 218 mg.day^-1^ (~900-3500 μmol.day^-1^) as estimated by the International Agency for Research on Cancer (IARC, 2010[35]). About 93 % of the total ingested nitrite is derived from nitrate reduction in the saliva, and only 7 % comes directly from food (Archer, 2002[1]). Endogenous and exogenous nitrate contributes about 1000 μmol.day^-1^ (37 %) and 1700 μmol.day^-1^ (63 %) in the total nitrate influx in the human body, indicating the higher contribution of the exogenous source.

### Absorption of nitrate and nitrite 

After ingestion, nitrate is primarily absorbed in the upper portion of the small intestine (i.e., duodenum) (Tannenbaum, 1979[103]), and its absorption is very effective with the bioavailability of around 100 % (van Velzen et al., 2008[112]). Nitrite also absorbs rapidly and effectively (90-98 %) from the gastrointestinal tract in humans (EFSA, 2008[22]; Hunault et al., 2009[33]). Nitrate cannot freely permeate through membranes and probably needs transporters (Ip et al., 2020[41]). Sialin (Qin et al., 2012[82]), AQP6 (Ikeda et al., 2002[39]), and CLC1 (Srihirun et al., 2020[99]) act as nitrate transporters, of which AQP6 is restricted to intracellular locations (Ikeda et al., 2002[39]), and CLC1 is exclusively present in the skeletal muscle (Conte et al., 2020[20]; Srihirun et al., 2020[99]). Silain is widely expressed in almost all tissues (Reimer, 2013[83]), and plasma membrane-targeted sialin can act as a 2NO_3_^-^/H^+^ cotransporter in the salivary glands (Qin et al., 2012[82]). However, the underlying mechanism of nitrate and nitrite absorption has not been determined. 

### Enterosalivary circulation of nitrate 

One hour after ingestion, the nitrate concentration in the saliva reaches a peak (Packer and Leach, 1991[77]). Nitrate entering the mouth during the first pass has a short residence time (Packer and Leach, 1991[77]). However, enterosalivary circulation of nitrate, recycling ingested nitrate via plasma to salivary nitrate (Archer, 2002[1]), extends the contact time of the oral cavity with dietary nitrate (Packer and Leach, 1991[77]). According to the elegant work of Spiegelhalder, of ingested nitrate, about 20 % (18.3±2.9 %) is secreted into the saliva (Spiegelhalder et al., 1976[98]). However, nitrate extraction by saliva is not fixed and values of 13-24 % (Spiegelhalder et al., 1976[98]), 20-25 % (Mortensen et al., 2017[72]; van den Brand et al., 2020[109]), and 20-28 % (Lidder and Webb, 2013[55]) of ingested nitrate have also been reported. 

Nitrate is reduced to nitrite by anaerobic bacteria on the tongue's surface (Benjamin et al., 1994[7]). The reduction of nitrate to nitrite also varies between individuals, and values between 5-36 % (Mortensen et al., 2017[72]) and 22-87 % (Packer and Leach, 1991[77]) have been reported. Based on large within and between-subject variations for both secretion rate of nitrate into the saliva (20-25 %) and conversion of nitrate to nitrite in the mouth (5-36 %), EFSA (European Food Safety and Authority) has estimated the overall conversion percentage of nitrate to nitrite to be between 1-9 % and proposed 5-7 % of the ingested nitrate for healthy adults (EFSA, 2008[22]; Mortensen et al., 2017[72]), which is consistent with another report (6.3 mol %.day^-1^) (Stephany and Schuller, 1980[100]). After overnight fasting, the mean salivary nitrite concentration is 114 μM and raised about 9 folds (1030 μM) 45 min following 200 mg potassium nitrate ingestion (Benjamin et al., 1994[7]). Antibacterial mouthwash use before ingestion of nitrate (10 mg.kg^-1^) in healthy volunteers reduced salivary nitrite by 77.6 % (514 μM immediately before nitrate ingestion compared with 115 μM 30 min after nitrate ingestion) (Jansson et al., 2008[42]). In the absence of dietary nitrate intake (absence of enterosalivary nitrate circulation), the amount of nitrite present in the saliva of adults is about 80 μmol.day^-1^ (Stephany and Schuller, 1980[100]), which can be attributed to nitrate produced from NOS-dependent NO pathway. It means that nitrate in the saliva is almost equally originated from ingested nitrate and NOS-derived nitrate.

Considering that 25 % of ingested nitrate is taken up from plasma by the saliva and of which 20 % is converted to nitrite in the oral cavity, a nitrate to nitrite conversion factor of 5 % (mol.mol^-1^) has been generally accepted (Archer, 2002[1]; Gangolli et al., 1994[26]; Kobayashi et al., 2015[48]; Larsson et al., 2011[51]). However, using a fixed conversion factor may lead to underestimating or overestimating nitrite exposure (van den Brand et al., 2020[109]). The proportion of ingested nitrate that is converted to nitrite is 5 % (mol.mol^-1^) for normally responding subjects (EFSA, 2008[22]; Joint FAO/WHO Expert Committee on Food Additives, 1995[44]) but can be as low as 1 % (Mortensen et al., 2017[72]) to as high as 20 % (EFSA, 2008[22]; Joint FAO/WHO Expert Committee on Food Additives, 1995[44]) or 22 % (Packer and Leach, 1991[77]) in different individuals. Intra- and inter-individual variabilities of bacterial species involved in nitrate-reducing in the oral cavity are 35-132 % and 27-120 %, respectively (Liddle et al., 2019[56]). Subjects, therefore, are categorized as low, medium, and high nitrate converters (Packer and Leach, 1991[77]), which convert 1-5 %, 5-10 %, >10 % of the ingested nitrate to nitrite, respectively. We showed that type 2 diabetic patients with higher salivary nitrate reductase activity have a more favorable metabolic response to nitrate supplementation (Bahadoran et al., 2021[5]).

In addition to interindividual differences (EFSA, 2008[22]; Joint FAO/WHO Expert Committee on Food Additives, 1995[44]), the amount of ingested nitrate (Spiegelhalder et al., 1976[98]) and aging (Brubacher et al., 2000[12], Torregrossa et al., 2011[106]; Vaiserman et al., 2017[108]) are other factors affecting the secretion of nitrate into saliva and its reduction to nitrite. The amount of secreted nitrate into the saliva is directly correlated with ingested nitrate (Spiegelhalder et al., 1976[98]). With an increasing amount of ingested nitrate, the percent of increases in salivary nitrate increases (if 100 mg nitrate is consumed, 12.4 mg (12.4 %) is extracted by the saliva, whereas if 500 mg is ingested 101.9 mg (20.4 %) is extracted (Spiegelhalder et al., 1976[98]). In addition, the amount of nitrite produced from nitrate in the saliva is directly correlated with the amount of nitrate ingested (Spiegelhalder et al., 1976[98]). 

NO production from the nitrate-nitrite-NO pathway is decreased in aging because of reduced gastric acid secretion (Torregrossa et al., 2011[106]), changes in oral and gut microbiota (Vaiserman et al., 2017[108]), and decreased ascorbic acid availability (Brubacher et al., 2000[12]). The stomach acid production decreases with age, and many people of advanced age are prescribed proton pump inhibitors (Torregrossa et al., 2011[106]). In addition, older subjects need more vitamin C intake for having a given plasma vitamin C concentration (Brubacher et al., 2000[12]) and experience changes in both gut and oral microbiota (Vaiserman et al., 2017[108]); all of them decrease the conversion of nitrate to nitrite to NO.

### The nitrogen cycle in the bacteria 

According to a revisited estimation, the human body contains 3.8×10^13^ bacteria with about 200 g wet weight, constituting approximately 0.3 % of the 70 kg reference man (Sender et al., 2016[90]). Bacteria are found in the gastrointestinal tract, skin, saliva, oral mucosa, and conjunctiva (Sender et al., 2016[90]), with the gut (Rocha and Laranjinha, 2020[85]), and particularly the colon (Sender et al., 2016[90]) having the highest number of the bacteria. *Firmicutes* and *Bacteroidetes* are the most abundant bacterial phyla in humans (Million et al., 2013[65]). Species in phyla *Firmicutes*, *Proteobacteria*, *Bactroidetes*, and *Actinobacteria* constitute >90 % of the oral microbiome (Koch et al., 2017[49]).

**Figure 2:**
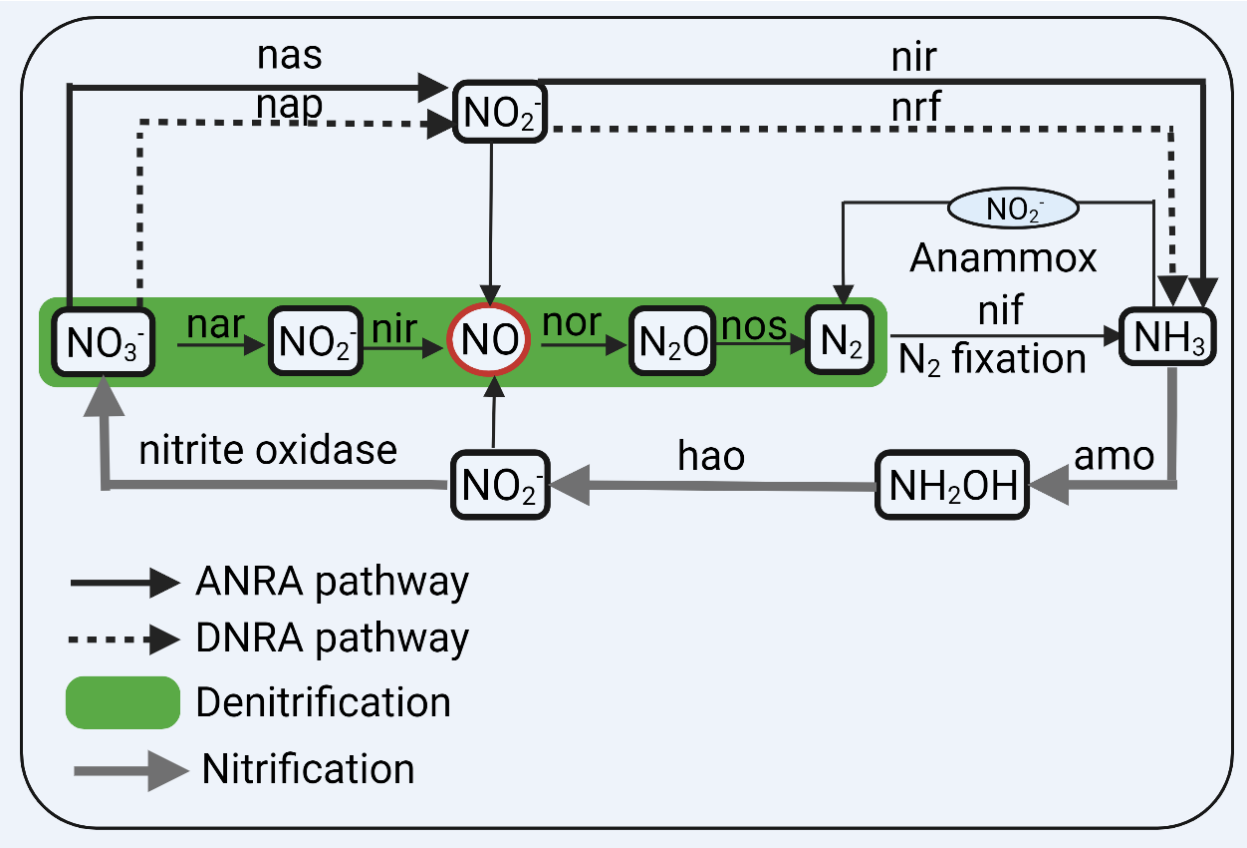
The nitrogen cycle in bacteria. Nitrate (NO_3_^-^) is converted to ammonia (NH_3_) via ANRA (assimilatory nitrate reduction to ammonia), DNRA (dissimilatory nitrate reduction to ammonia), and denitrification pathways. Dinitrogen (N_2_) fixation converts N_2_ to NH_3_. NH_3_ is converted to NO_3_^-^ aerobically (nitrification) or anaerobically via the anammox (anaerobic ammonium oxidation) process. nas, nap, nar are nitrate reductases, and nir and nrf are nitrite (NO_2_^-^) reductases. amo, ammonia oxidase; hao, hydroxylamine oxidoreductase; N_2_O, nitrous oxide. Created with BioRender.com

Bacteria use nitrate and nitrite as electron acceptors in their respiration (Rocha and Laranjinha, 2020[85]; Sparacino-Watkins et al., 2014[97]). Nitrate is the most stable and important source of nitrogen compounds (Sparacino-Watkins et al., 2014[97]). In the bacterial nitrogen cycle (Figure 2(Fig. 2)), NO_3_^-^ is the most oxidized form (oxidation state=+5), and ammonia (NH_3_) is the most reduced form of nitrogen (oxidation state=-3) (Sparacino-Watkins et al., 2014[97]). Reduction of nitrate by bacteria is achieved by three major pathways (Koch et al., 2017[49]; Sparacino-Watkins et al., 2014[97]) (Figure 2(Fig. 2)): (1) two-step assimilatory nitrate reduction to ammonia (ANRA), (2) two-step dissimilatory nitrate reduction to ammonia (DNRA), and (3) four-step denitrification; these pathways are mainly involved in the production of bacterial biomass, detoxification of nitrogen compounds, and ATP generation, respectively. Note that the fate of reduced nitrogen determines assimilatory or dissimilatory nitrate reduction; if incorporated into the biomass, it is called assimilatory, and if it is excreted from the cell, it is called dissimilatory (Sparacino-Watkins et al., 2014[97]). 

Nitrate reduction to nitrite in the ANRA, DNRA, and denitrification pathways are made by cytoplasmic (nas), periplasmic (nap), and membrane-bound (nar) nitrate reductases, respectively (Koch et al., 2017[49]). In the ANRA and DNRA pathways, nitrite is converted to NH_3_ by nitrite reductases (nir and nrf), respectively (Koch et al., 2017[49]). In the denitrification pathway, nitrite is reduced to NO by nir, NO is reduced to nitrous oxide (N_2_O) by nor (NO reductase), and N_2_O is reduced to dinitrogen (N_2_) by nos (nitrous oxide reductase); nitrogen fixation reduces N_2_ to NH_3_ by nif (nitrogenase) (Koch et al., 2017[49]).

Oxidation of NH_3_ is done both in the aerobic (nitrification) and anaerobic (anaerobic ammonium oxidation = anammox process) conditions and anammox (free energy = - 357 kJ.mol^-1^) is energetically more favorable than nitrification (free energy = - 275 kJ.mol^-1^) (Jetten et al., 2001[43]). Anammox and nitrification oxidize NH_3_ to NO_3_^-^ and N_2_, respectively (Sparacino-Watkins et al., 2014[97]). 

### Reduction of nitrite to NO in the stomach

Nitrate in the stomach has three sources: (1) ingested dietary nitrate, (2) enterosalivary circulation of nitrate, and (3) nitrate secretion from circulation (Packer and Leach, 1991[77]). Normal subjects' intragastric nitrate levels are around 108-520 μM and may exceed the saliva's (Packer and Leach, 1991[77]). In the normal acid stomach (pH range: 1-3), intragastric nitrite is mostly of the salivary origin (Iijima et al., 2003[37]; Packer and Leach, 1991[77]). Intragastric nitrite levels in normal subjects are around 3 μM (Packer and Leach, 1991[77]). Nitrite concentration affects NO production in the stomach (Benjamin et al., 1994[7]) with increasing nitrite concentrations increases NO production (Iijima et al., 2003[37]). NO concentration in expelled air from the stomach of healthy non-smoker subjects was reported to be about 20 nM and increased fourfold after 50 g lettuce (Lundberg et al., 1994[62]). It has been proposed that nitrite has gastric absorption as NO_2_^-^ or nitrous acid (HNO_2_) (Licht et al., 1986[54]) while nitrate has little gastric absorption (Packer and Leach, 1991[77]). 

Acidification of nitrite in the stomach produces HNO_2_ (pKa=3.2), which is converted to nitrosating species [dinitrogen trioxide (N_2_O_3_) and nitrosonium (NO^+^)] and then to NO (Benjamin et al., 1994[7]; Iijima et al., 2003[37]), which can diffuse through cell membranes (Benjamin et al., 1994[7]). pKa is the pH at which the acid is 50 % dissociated (Butler and Feelisch, 2008[15]). In fact, at pH <3 and in the presence of ascorbic acid, most nitrite is converted to NO in a few seconds (Iijima et al., 2003[37]). NO production from salivary nirite in humans is maximal at gastroesophageal junction and cardia of the stomach (Iijima et al., 2002[38]). 

Two main factors in converting nitrite to NO in the stomach are acidic juice (Benjamin et al., 1994[7]; Lundberg et al., 1994[62]), which converts NO_2_^-^ to HNO_2_, and ascorbic acid, which converts HNO_2_ to NO (Iijima et al., 2003[37]). In fasted humans, intragastric pH is about 1.5, and gastric juice's mean ascorbic acid concentration is 100 μM (range 10-300 μM) (Iijima et al., 2003[37]). Pretreatment with omeprazole, which inhibits gastric acid secretion, significantly decreases NO concentration in the expelled air from the stomach in both the presence and absence of lettuce (a source of nitrate) intake (Lundberg et al., 1994[62]). Based on the simulation of the gastric environment, it has been reported that a very low level of NO is produced in the presence of pH>3 (Lundberg et al., 1994[62]). For conversion of nitrite to NO, in addition to acidic pH, ascorbic acid is required, and the amount of NO generation is increased dose-dependently with increasing nitrite and ascorbic acid (Okazaki et al., 2006[74]). In addition, very little NO is formed in the absence of ascorbic acid even at low pH (1.5) (Iijima et al., 2003[37]). These findings indicate the necessity of both an acidic environment and ascorbic acid for the optimal conversion of nitrite to NO in the stomach. 

Following nitrate ingestion, there is no rise in gastric nitrite concentration but administration of omeprazole (40 mg.day^-1^, 4 weeks), which decreases the conversion of nitrite to NO, increased gastric nitrite level following nitrate ingestion (Mowat et al., 1999[73]). These data indicate that converting stomach nitrite to NO in healthy subjects is a rapid process so that about 80 % of the peak NO concentration is observed within 10 seconds (Iijima et al., 2003[37]; Mowat et al., 1999[73]). However, the rate of decline of NO concentrations in the stomach (1-5 min for fall by 50 %) is much slower than the rate of NO production (Iijima et al., 2003[37]). In these conditions, NO production in the stomach from nitrite provides a continuous formation of NO (Lundberg et al., 1994[62]).

### NO production in the intestine 

In addition to the passage of nitrate down the gut (Witter et al., 1979[119]), secretion of nitrate into the small (Witter et al., 1979[118][119]) and large (Packer and Leach, 1991[77]) intestines and intestinal nitrification (Figure 2(Fig. 2)) (Gomez et al., 1980[31]; Tannenbaum, 1979[103]) may contribute to the presence of nitrate in the lower intestinal tract. In conventional-flora rats, following nitrate ingestion, nitrate was detected in the stomach, small intestine, and large intestine (Witter and Balish, 1979[117]), and up to 24 % of gavaged ^13^N from ^13^NO_3_^-^ can reach the lower intestinal tract (i.e., the cecum and large intestine) (Witter et al., 1979[119]). Experiments in pigs have shown that following *L*-N^G^-nitro arginine methyl ester (*L*-NAME) administration, luminal NO levels in the small intestine decreases by ~90 % from 879 to 88 μM; however, no change was observed for nitrate and nitrite, indicating that NO in the intestinal lumen is NOS-dependent and that source of nitrate and nitrite in the intestinal lumen is NOS-independent (Eriksson et al., 2018[23]). After nitrate injection (10 mg.kg^-1^) to pigs, luminal concentrations of both nitrate and nitrite in the small intestine were increased even in the presence of NOS inhibition, indicating secretion of nitrate to the small intestine lumen and its conversion to nitrite (Eriksson et al., 2018[23]). The presence of nitrite but not nitrate in fecal and ileostomy samples in humans indicates that nitrification of ammonia or organic nitrogen compounds occurs in the upper, aerobic portion of the intestine (Tannenbaum et al., 1978[103]). 

There is some evidence that enteric bacteria can produce NO; enteric bacteria may produce NO via ANRA (Tiso and Schechter, 2015[105]), DNRA (Tiso and Schechter, 2015[105]; Vermeiren et al., 2009[113]), and denitrification (Tiso and Schechter, 2015[105]) pathways with the DNRA pathway seems to be the most important pathway in the colon (Vermeiren et al., 2009[113]). It has been estimated that bacterial metabolism in the lower GI tract may contribute to the metabolism of 20 % of ingested nitrate (Packer and Leach, 1991[77]). It has been shown that in intubated and anesthetized pigs, when enterosalivary circulation of nitrate is greatly decreased, nitrate injection (10 mg.kg^-1^) can counteract the decline in circulating nitrite observed in *L*-NAME-treated animals, indicating nitrate to nitrite conversion in other locations apart from the oral cavity, most probably in the small intestine lumen (Eriksson et al., 2018[23]). In addition, NO levels in the caecum of germ-free rats increase by 10-fold following colonization with a normal bacterial flora (9 vs. 93 ppb) (Sobko et al., 2005[94]).

### Nitrate reduction by eukaryotic cells 

Two hours after sodium nitrate (10 mg.kg^-1^) gavage to germ-free mice, plasma nitrite levels were increased, indicating that mammalian tissue can reduce nitrate to nitrite *in vivo* and in normoxic conditions (Jansson et al., 2008[42]). Addition of nitrite (100 μM) causes NO production in the liver (~0.7 nM.s^-1^), heart (~0.5 nM.s^-1^), and aorta (~0.3 nM.s^-1^) of Sprague-Dawley rats (Li et al., 2008[52]). In Sprague-Dawley rats and under anaerobic conditions, the rate of NO formation from nitrite (100 μM) in the presence of NOS inhibition was 0.78, 0.58, and 0.004 nM s^-1^, in the liver, heart, and blood, respectively (Li et al., 2008[52]); when the percent of oxygen was increased to 10 %, these values were decreased by 77 %, 81 %, and 83 %, respectively (Li et al., 2008[52]). These findings indicate that tissues produce NO from nitrite in normoxia condition that increased in hypoxic condition and under both conditions, NO is produced in tissues rather than in blood (Jansson et al., 2008[42]; Li et al., 2008[52]). 

Combined inhibition of xanthine oxidase (XO) and aldehyde oxidase (AO) decrease NO generation from nitrite by more than 65-70 %, indicating the major contribution of these enzymes in the reduction of nitrite to NO with only 15-20 % remaining for non-enzymatic reduction of nitrite (Li et al., 2008[52]). Molybdenum-containing enzymes (XO and AO) are found in the lung, blood vessels, heart, and kidney (Zweier et al., 2010[121]). Xanthine oxidoreductase (XOR) is a mammalian enzyme with structural similarity with bacterial nitrate reductases (Jansson et al., 2008[42]). Mammalian nitrate reductase activity is most abundant in the gut and liver with the order of colon>stomach>kidney>small intestine> liver>heart>lung (Jansson et al., 2008[42]).

Nitrite reduction to NO can be done via enzymatic pathways (including XOR, AO, deoxygenated hemoglobin and myoglobin, cytochrome P_450_, cytochrome c, eNOS, and mitochondrial respiratory chain) or spontaneously in an acidic environment (disproportionation) (Jansson et al., 2008[42]; Omar and Webb, 2014[75]; van Faassen et al., 2009[110]; Zweier et al., 2010[121]). Disproportionation is limited to the stomach and ischemic tissues because of low pKa of nitrite (i.e., 3.4) (Gladwin et al., 2005[30]). NO production rate from nitrite disproportionation in normal tissues with pH 7.2 to 7.4 and nitrite concentration of 10 to 50 μM is 0.05-1 pM.s^-1^ (0.05-1 fmol.s^-1^.g^-1^) (Samouilov et al., 1998[87]) that is equal to about 6 μmol.day^-1^ in a 70-kg man. Under ischemic conditions, nitrite disproportionation can increase to 4-100 pM.s^-1^ but is still 5-10 % of the maximum NOS-dependent NO production under physiological state (Samouilov et al., 1998[87]). A decrease in pH increases NO production from nitrite; in the presence of nitrite (20 μM), one unit decrease in pH from 7.0 to 6.0 increased NO generation by about 12 and 13 folds in the liver and heart tissues, respectively (Li et al., 2008[52]). Thus, non-enzymatic reducing nitrite to NO is crucial in ischemic conditions in which both hypoxia and acidic pH are present.

## Conclusion and Perspectives

About 90 % of the whole-body NO production can be attributed to the NOS-dependent *L*-arginine-NO pathway. The nitrate-nitrite-NO pathway contributes about 10 % to the whole-body NO production. The lower contribution of the nitrate-nitrite-NO pathway does not mean that this pathway has lower importance in the whole-body NO homeostasis. Supports for this notion are decreased skeletal muscle nitrate and nitrite in rats (Gilliard et al., 2018[29]) and developing metabolic syndrome and endothelial dysfunction in mice (Kina-Tanada et al., 2017[47]) following deprivation or lowering dietary nitrate and nitrite. Indeed, it seems that the nitrate-nitrite-NO pathway is complementary to the NOS-dependent NO production and not only a backup system, as it is mostly presumed. Thus, NOS-dependent NO production is insufficient to meet all NO functions even in the adequate *L*-arginine supply and normoxic conditions, and the nitrate-nitrite-NO pathway must provide the rest (Gilliard et al., 2018[29]). These two pathways cross-talk each other. In eNOS^-/-^-deficient mice nitrite reductase activity increased by about 90 % in the liver, suggesting a compensatory mechanism for enhancing NO production in the absence of NOS (Bryan et al., 2008[14]). However, nitrate and nitrite transport mechanisms across cell membranes have not been completely understood. 

Some points deserve further consideration regarding evidence for assessing the quantitative aspects of NO production. First, the average rates of NOS-dependent NO synthesis reported in different studies differ by 2-fold using different methods for quantifying NO production (Siervo et al., 2011[92]). In the nitrate-nitrite-NO pathway, there is significant inter-individual variability in both nitrate extraction by saliva (13-28 %) and the conversion of nitrate to nitrite (5-36 %), yielding very different efficiency for NO production from a given amount of nitrate-containing food. These issues make the comparison between studies complex and should be considered in interpreting the results. 

Second, species difference is a factor that may explain some inconsistent findings in the field. Rodents are the most common species used in animal experiments (Ghasemi et al., 2021[28]). In rats, NO production rate is similar to humans (0.55 ± 0.05 vs. 0.38±0.06 μmol.kg^-1^.h^-1^ (Siervo et al., 2011[92]). However, on a body weight basis, the whole-body NOS-dependent, NO production in mice is about 20 times higher than in humans (7.68 ± 1.47 vs. 0.38 ± 0.06 μmol kg^-1^ h^-1^) (Siervo et al., 2011[92]). In addition, the amount of nitrate and nitrite in rodent diet is reported to be considerably different [340.3±13.5 nmol.g^-1^ and 7.4±0.1 nmol.g^-1^ (Gilliard et al., 2018[29]; Park et al., 2021[80]) in NIH standard rodent diet and 6275 ± 50.7 and 104.3 ± 4.7 nmol.g^-1^ in Purina rodent chow (Bryan et al., 2008[14]), for nitrate and nitrite, respectively], indicating more than 10-fold difference. Unlike humans, rats and mice do not effectively concentrate nitrate in the saliva (Montenegro et al., 2016[69]), and it seems that other nitrate-reducing mechanisms such as XOR may act in parallel with nitrate-reducing bacteria to control NO signaling (Montenegro et al., 2016[69]). In addition, the accumulation of intravenously-injected ^13^NO_3_ in the salivary gland of humans (4 %) is 10 times that in rats (0.4 %) (Witter et al., 1979[118]). On the other hand, it has been argued that nitrate reduction in basal conditions is more effective in rodents than in humans but, humans, unlike rodents, can accumulate nitrate in the saliva with a concentration 10-20 fold higher than blood and therefore overall capacity to produce nitrite from nitrate is comparable between different mammalian species (Jansson et al., 2008[42]).

Last but not least, there is a possibility for NOS- and NO_3_^-^-independent NO production; based on the observation in mice and rats that following combined low nitrate and nitrite diet and NOS inhibition or knockdown, protein-bound NO modifications still persist, Milsom et al. provided evidence that there is another source of NO formation other than the *L*-arginine-NO and nitrate-nitrite-NO pathways (Milsom et al., 2012[66]). However, the duration of the Milsom et al. study (3 h in mice and 2 weeks in rats) seems to be insufficient for this conclusion since following the switch from high to low-level nitrate ingestion, outflow from tissues may persist for several days (Packer and Leach, 1991[77]). The total body content of nitrate has been estimated to be about 0.41 g (6700 μmol) (Packer and Leach, 1991[77]) or 1 g (~16000 μmol) (Witter et al., 1979[118]) in humans, which is about 6 to 15 times of average daily NO synthesis by both NO production pathways. 

## Declaration

### Conflict of interest 

The author declares that he has no competing interests.

### Acknowledgments 

Shahid Beheshti University of Medical Sciences supported this study (Grant No. 31982-1).

## Figures and Tables

**Table 1:**
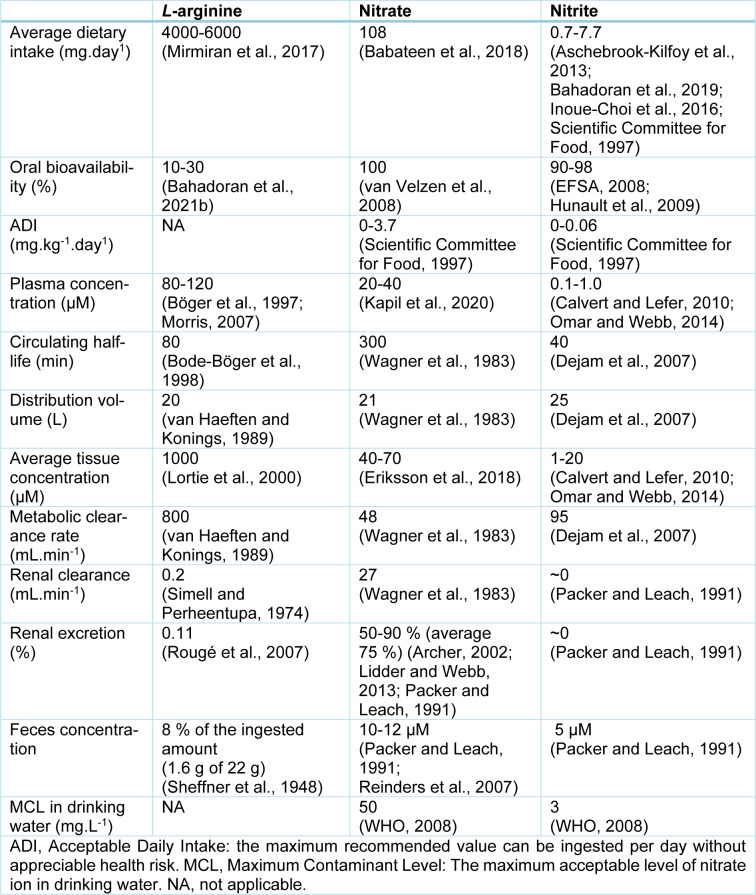
Summarized data on *L*-arginine, nitrate, and nitrite kinetics
